# Investigating the predictive factors of thoracic aortic invasion and surgical outcomes in patients with primary lung cancer: A retrospective study

**DOI:** 10.1111/1759-7714.15311

**Published:** 2024-04-16

**Authors:** Hideomi Ichinokawa, Kazuya Takamochi, Mariko Fukui, Aritoshi Hattori, Takeshi Matsunaga, Kenji Suzuki

**Affiliations:** ^1^ Department of General Thoracic Surgery Juntendo University Hospital Tokyo Japan

**Keywords:** computed tomography, extended surgery, lung cancer, T4, thoracic aortic invasion

## Abstract

**Background:**

This study aimed to investigate predictors of thoracic aortic invasion in lung cancer patients using preoperative clinical and imaging characteristics and elucidate surgical outcomes in cases of aortic invasion.

**Methods:**

Of the 4751 lung cancer patients who underwent surgery at our hospital, we included 126 (6.8%) who underwent left‐sided surgery and in whom tumor appeared to be in contact with the thoracic aorta on preoperative imaging. The patients were divided into two groups: group A, 23 patients (18%) who underwent combined aortic resection (+); group B, 103 patients (82%) who did not undergo combined aortic resection (−).

**Results:**

The percentage of aortic invasion for tumor diameter <3 cm, 3–4 cm, 4–5 cm, 5–7 cm, and >7 cm was 0%, 13%, 23%, 16%, and 35%, respectively. The percentages of aortic invasion were 27%, 16%, and 0% for tumor localization in the upper division, S6, and S10, respectively. Multivariate analysis revealed that aortic depression due to tumor or loss of fatty tissue between tumor and mediastinum in the chest CT significantly predicted aortic invasion (odds ratio = 23.83, 16.66). Group A demonstrated significantly more blood loss, longer operative time, prolonged hospital stay, and increased percentage of recurrent nerve palsy (13%) compared to group B. The 1‐, 3‐, and 5‐year survival rates for patients in group A were 53.4%, 24.3%, and 24.3%, respectively.

**Conclusion:**

If the chest CT of a patient demonstrates aortic depression due to tumor or loss of fatty tissue between tumor and mediastinum, aortic complications should be considered when planning surgery.

## Graphical abstract

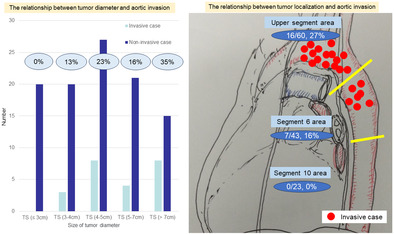

## INTRODUCTION

Surgical resection is the cornerstone for the treatment of non‐small cell lung cancer. In recent years, with advances in surgical techniques, instruments, intra‐ and postoperative management, and appropriate case selection, extended surgery for primary lung cancer has received increasing attention. However, the prognosis of patients with T4 lung cancer invading the aorta has been reported to be poor even if complicated resection or reconstruction is performed.^1^, ^2^ Studies have reported that long‐term survival can be expected in some cases.^3^, ^4^ A study reported that long‐term survival can be expected in N0 cases.^4^ However, the number of patients with primary lung cancer demonstrating aortic invasion is relatively less. Moreover, only a few studies have investigated the preoperative predictors of aortic invasion in patients with primary lung cancer,^5^, ^6^, ^7^ and the number of cases included in the studies was small. Furthermore, if the presence of aortic invasion could be predicted preoperatively, it would be good for medical economics, such as eliminating the need for cardiovascular surgeons and artificial heart‐lung standby.

Therefore, in this study, we investigated the preoperative clinical and imaging features to identify the predictors of thoracic aortic invasion. We also aimed to determine the surgical outcomes of thoracic aortic invasion.

## METHODS

### Study population

This retrospective study was approved by the Ethics Committee of Juntendo University Hospital (approval no.: E23‐0325) and performed in accordance with the tenets of the Declaration of Helsinki. The need for informed consent was waived by the Ethics Committee due to the retrospective nature of the study.

**FIGURE 1:**
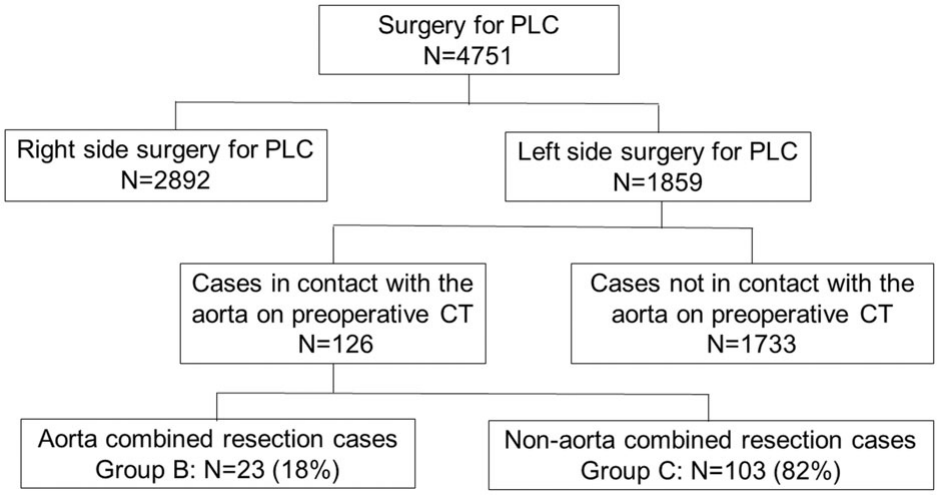
Patient distribution in the study.

A total of 4751 patients underwent surgeries for lung cancer at our institution between August 2006 and December 2021. Among them, 126 patients (6.8%) who underwent left‐sided surgery and in whom, the tumor appeared to be abutting the thoracic aorta on preoperative chest computed tomography (CT) images formed our study population. The selected patients were then divided into two groups: group A comprising 23 patients (18%) with aortic invasion and group B comprising 103 patients (82%) without aortic invasion (Figure 1). The following clinical background characteristics along with the peri‐ and postoperative results were analyzed: age, sex, chief complaint, preoperative comorbidities, preoperative treatment, smoking history, pack‐year smoking, respiratory function (vital capacity [VC], %VC, forced expiratory volume in 1 s [FEV 1.0], FEV 1.0%), tumor size, clinical stage, pathological findings, surgical procedures, duration of surgery, intraoperative blood loss, hospital stay, and Clavien–Dindo grade ≥ 2 postoperative complications. Surgical morbidity and mortality rates within 30 and 90 days of the surgery were also evaluated.

### Preoperative staging

**FIGURE 2:**
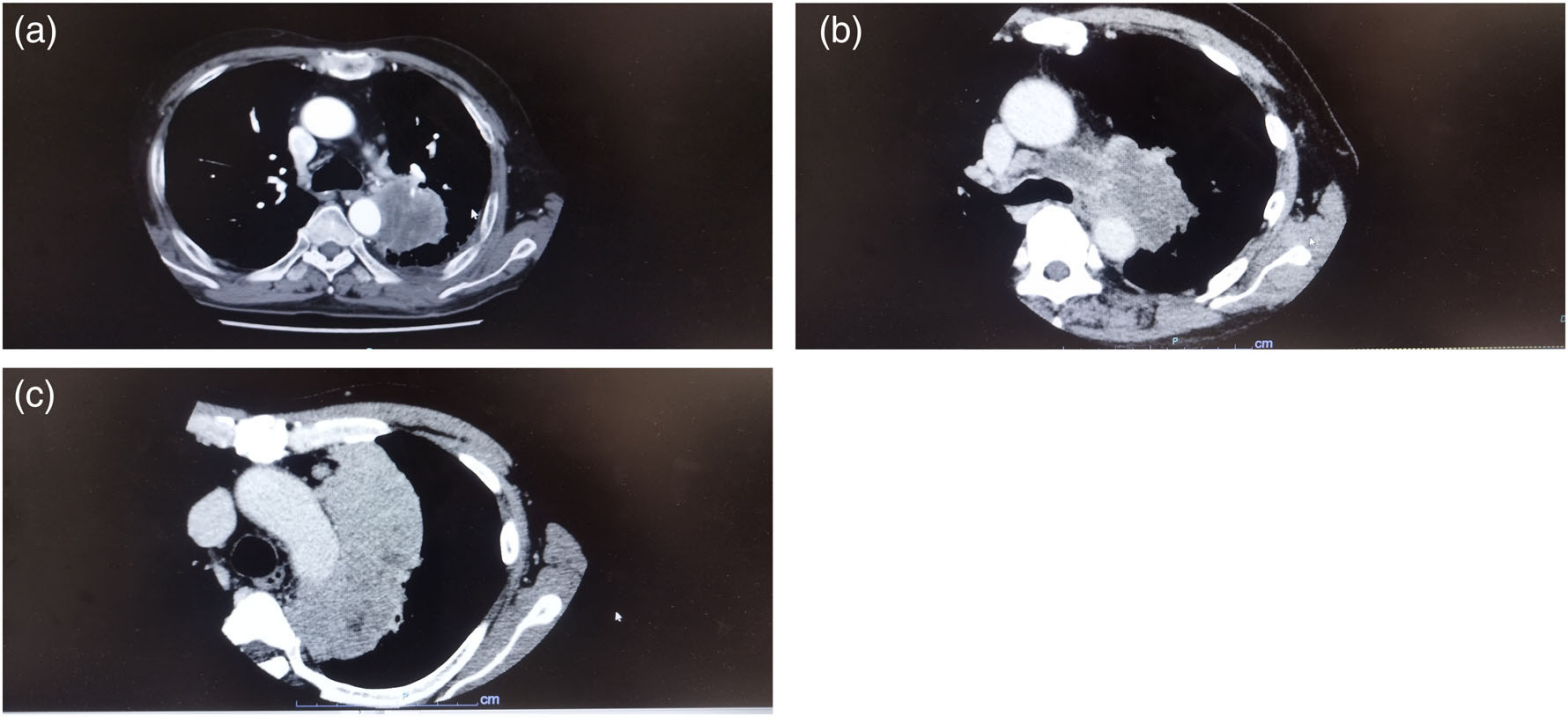
Chest computed tomography (CT) findings due to the tumor. (a) Approximation to the aorta by >90°. (b) Aortic depression. (c) Loss of fatty tissue between tumor and mediastinum.

All patients underwent preoperative axial CT scan of the thorax with a slice thickness of ≤3 mm and mediastinal (level, 40 Hounsfield units [HU]; width, 400 HU) and lung (level, 600 HU; width, 1600 HU) window settings to evaluate the primary tumor and mediastinal nodes. In addition, head CT or head magnetic resonance imaging (MRI) was performed along with positron emission tomography (PET)‐CT for full‐body examination. If mediastinal node involvement was suspected, the mediastinum was assessed using endobronchial ultrasound‐guided fine‐needle aspiration. For our imaging findings, we reviewed all the 2 mm thin slice CT images from each case and decided via consensus. We investigated the presence or absence of the following four findings: (1) approximation to the aorta by >90°, (2) contact with the aorta by a major diameter of ≥30 mm, (3) aortic depression, and (4) loss of fatty tissue between the tumor and mediastinum on chest CT (Figure 2a–c). To determine the angle between the aorta and the tumor, we determined the image that was most tangential between the two. Next, we measured the contact angle of the center of the aorta on its axis with the tumor to determine if they were in contact by >90°. We defined loss of fatty tissue between the tumor and mediastinum as a loss of ≥10 mm of contiguous fatty tissue between the aorta and the tumor in the mediastinal imaging condition. Tumors were classified according to the eighth edition of the TNM classification system of malignant tumors.^8^

### Operative procedure and follow‐up

The surgical procedure was determined based on the extent of the disease. Although a lobectomy was preferred, segmentectomy, bilobectomy, sleeve resection, or pneumonectomy was performed, if required, due to the location of the primary tumor or metastatic nodal invasion. All patients underwent complete ipsilateral mediastinal and subcarinal nodal dissection. Group A (*n* = 23) comprised 13 cases of R0 with combined resection of aortic adventitia, six cases of R1 and R2 resection due to invasion into the aortic media, and four cases of artificial vessel replacement due to invasion into the aortic media.

Follow‐up information on all the patients was obtained through office visits or telephonic interviews. The postoperative follow‐up protocol was as follows. Patients were evaluated every 3 months by physical examination, chest roentgenography, analysis of blood chemistry variables, and measurements of the tumor marker levels. Chest and abdominal CT or PET‐CT were repeated every year for 5 years. The median observation period from the time of first surgery was 977 days.

### Statistical analysis

Descriptive statistics were used to assess the demographic characteristics of patients and outcomes. Normally distributed continuous data are expressed as median values, and categorical data expressed as counts and proportions. Survival was calculated using the Kaplan–Meier method, and differences in survival were assessed using log‐rank analysis. Comparisons among all parameters were analyzed using the student's *t‐*test. Multivariate analysis was performed by logistic regression analysis using the SPSS Statistics 21 software program (IBM Corporation). Forward and backward stepwise procedures were used to elucidate the significant factors that were essential for predicting aortic invasion. The level of statistical significance was set at *p* < 0.05.

## RESULTS

### Comparison of preoperative clinical features between aortic invasion and noninvasion groups (group A vs. group B)

Table 1 presents the preoperative clinical features of groups A and B. In group A, the median age of patients was 65 years, 22 (96%) were male, and 18 (78%) presented with a chief complaint. The chief complaints were back pain in six, cough in five, bloody sputum in five, and others in five patients. In group A, the median tumor diameter was 50 mm, 15 patients (65%) had clinical stage IIIA or higher, and 12 (52%) had squamous cell carcinoma as the common histological type. Compared to patients in group B, those in group A were predominantly males, presented with a chief complaint, comprised cases with clinical stage of IIIA or higher, and had a larger tumor size (*p* < 0.05). No significant differences were observed between the two groups in terms of age, preoperative comorbidities, preoperative treatment, smoking history, pack‐year smoking, respiratory function, and percentage of squamous cell carcinoma in pathology. Group A had a significantly higher proportion of positive chest CT findings for more than 90° approximation to the aorta, contact with the aorta with a major diameter of ≥30 mm, and loss of fatty tissue between tumor and mediastinum than group B (*p* < 0.05).

**TABLE 1:** Characteristics of the patients who underwent surgery for lung malignancy in whom chest computed tomography (CT) demonstrated the tumor to be adjacent to the aorta.

Variables	Group A	Group B	*p*‐value
	(*n* = 23)	(*n* = 103)	
Age, median [IQR]	65 [58–72]	70 [62–76]	0.38
Male sex	22 (96%)	63 (61%)	<0.05
Chief complaint, yes	18 (78%)	37 (36%)	<0.05
Preoperative comorbidity	17 (74%)	90 (87%)	0.12
Preoperative treatment, yes	5 (22%)	11 (11%)	0.17
Smoking history	20 (87%)	75 (73%)	0.19
Pack‐year smoking [IQR]	36.0 [32.0–56.3]	32.0 [0–52.0]	0.51
VC, L [IQR]	3.26 [2.99–3.59]	2.85 [2.36–3.75]	0.41
%VC, % [IQR]	87.8 [84.6–97.9]	91.8 [80.8–103.7]	0.69
FEV1, L [IQR]	2.22 [2.09–2.47]	1.96 [1.56–2.53]	0.41
FEVI%, % [IQR]	70.3 [68.9–76.3]	72.0 [65.7–77.4]	0.90
Tumor size, mm [IQR]	50 [45–77]	45 [32–60]	<0.05
Chest CT findings			
Approximation to the aorta by >90°	19 (83%)	43 (42%)	<0.001
Contacts the aorta with a major diameter of ≥30 mm	20 (87%)	52 (50%)	<0.05
Aortic depression	7 (30%)	1 (1%)	<0.001
Loss of fatty tissue between tumor and mediastinum	20 (87%)	25 (24%)	<0.001
Clinical stage			
I	3 (13%)	36 (35%)	
II	5 (22%)	35 (34%)	
IIIA or higher	15 (65%)	32 (31%)	<0.05^a^
Pathology			
Squamous cell carcinoma	12 (52%)	44 (43%)	
Adenocarcinoma	5 (22%)	48 (47%)	
Others	6 (26%)	11 (10%)	0.49^b^

Abbreviations: CT, computed tomography; FEV1, forced expiratory volume in 1 s; IQR, interquartile range; VC, vital capacity.

^a^ Clinical stage I, II versus IIIA or higher.

^b^ Squamous cell carcinoma versus nonsquamous cell carcinoma.

### Tumor diameter and tumor localization in relation to aortic invasion

**FIGURE 3:**
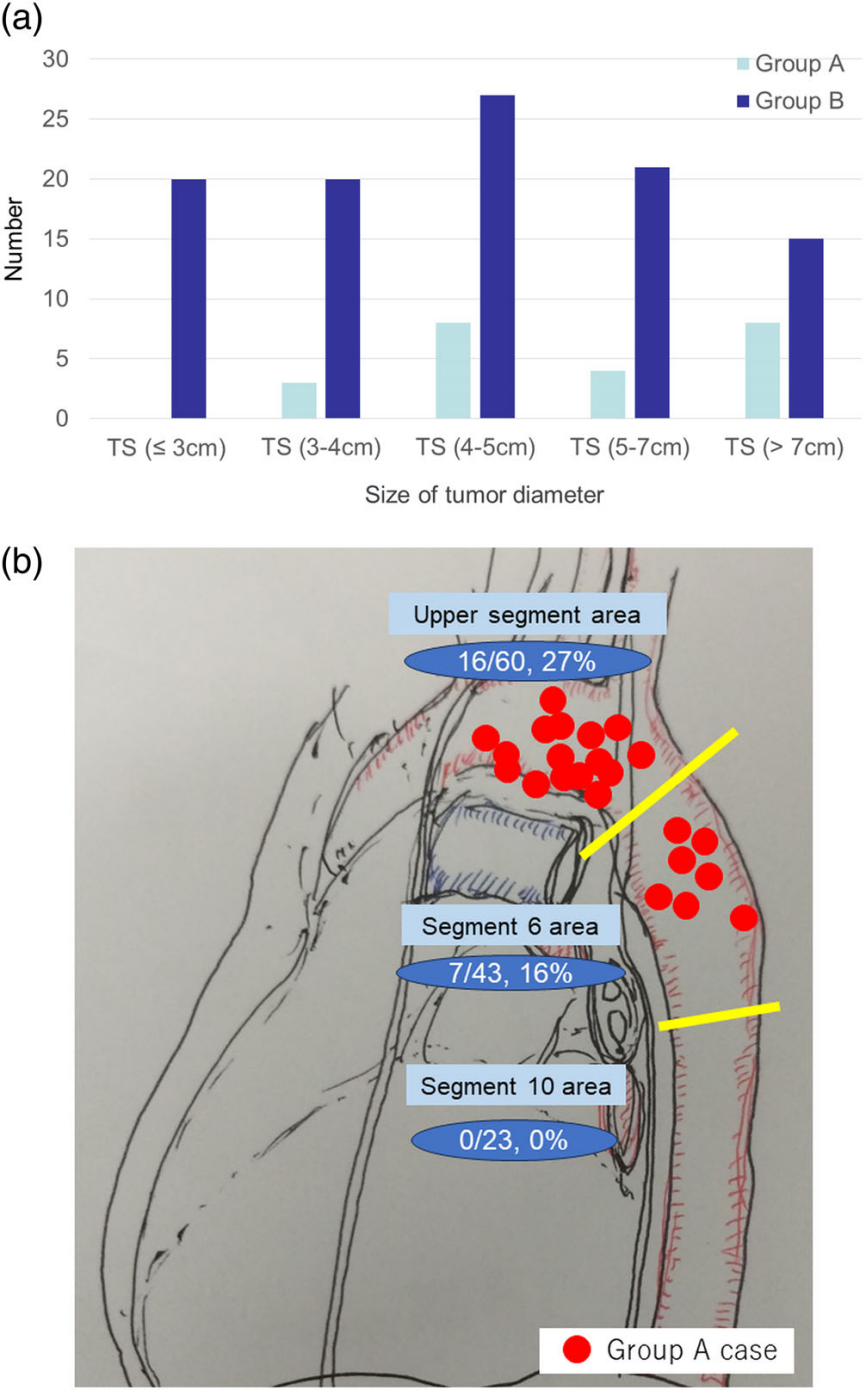
Differences in the degree of invasion depending on the size and location of the tumor. (a) The relationship between tumor diameter and aortic invasion and (b) the relationship between tumor localization and aortic invasion. TS, tumor size.

Figure 3a shows the relationship between tumor diameter and aortic invasion. None of the 20 cases with tumor diameters <3 cm demonstrated invasion. For tumor diameters of 3–4 cm, 4–5 cm, 5–7 cm, and >7 cm, 3/23 (13%), 8/35 (23%), 4/25 (16%), and 8/23 (23%) cases elicited invasion, respectively. With the increase in the tumor diameter, the tumor itself had a higher capacity to invade other organs. Figure 3b shows the relationship between tumor localization and aortic invasion. Tumor localization was categorized into upper division area, segment 6 area, and segment 10 area. In upper division area, 16/60 (27%) and in segment 6 area, 7/43 (16%) demonstrated aortic invasion. In segment 10, none of the 23 cases exhibited aortic invasion.

### Univariate and multivariate analyses of aortic invasion

According to the univariate analysis, male sex, patients reporting with a chief complaint, tumor diameter >3 cm, tumor localization other than segment 10, clinical stage IIIA or higher, an approximation to the aorta by >90°, contact with the aorta with a major diameter of ≥30 mm, aortic depression, and loss of fatty tissue between tumor and mediastinum were significant predictors of aortic invasion (*p* < 0.05) (Table 2). Multivariate analysis revealed that aortic depression due to the tumor or loss of fatty tissue between the tumor and mediastinum predicted aortic invasion (*p* = 0.039, 0.026).

**TABLE 2:** Cox proportional hazards models for prediction of aortic invasion (*N* = 126).

Variables	Univariate analysis	Multivariate analysis		*p*‐value
	*p*‐value	OR	95% CI	
Sex, male	<0.001			0.057
Chief complaint, yes	<0.001			0.10
Tumor size, >30 mm	0.023			0.90
Tumor localization, other than segment 10	0.012			0.89
Clinical stage: IIIA or higher	0.004			0.33
Approximation to the aorta by >90°	<0.001			0.85
Contacts the aorta with a major diameter of ≥30 mm	0.002			0.61
Aortic depression	<0.001	23.83	1.17–485.67	0.039
Loss of fatty tissue between tumor and mediastinum	<0.001	16.66	1.39–199.32	0.026

Abbreviations: CI, confidence interval; OR, odds ratio.

### Comparison of peri‐ and postoperative course (group A vs. group B)

**FIGURE 4:**
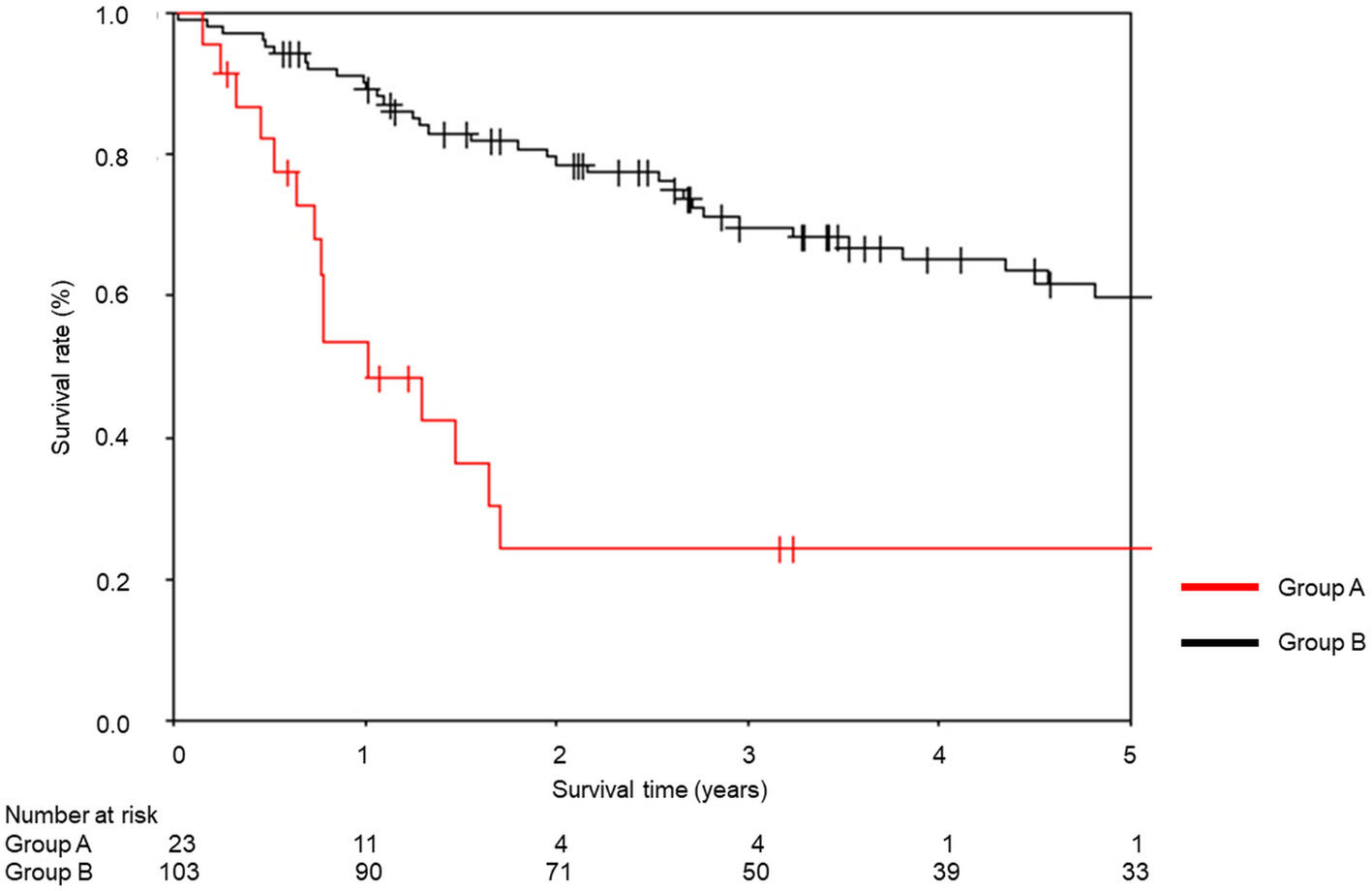
Kaplan–Meier survival curves of groups A and B demonstrating 5‐year overall survival.

Table 3 shows the peri‐ and postoperative course characteristics of group A and group B. Group A had a median operative time of 221 min, median blood loss of 180 mL, and a median hospital stay of 15 days. Group A had significantly longer operative time, more blood loss, and prolonged hospital stay than group B. A total of 14 patients (61%) in group A had postoperative complications. There was no significant difference between the two groups in terms of total postoperative complication rate. Postoperative complications in group A comprised pulmonary fistula in four patients (17%), arrhythmia in three (13%), recurrent nerve palsy in three (13%), chylothorax in two (9%), and others in two (9%). Group A had a higher prevalence of recurrent nerve palsy as a postoperative complication than group B (*p* < 0.05). In group A, none of the patients died within 30 days, while one (4%) died within 90 days. The patient developed an acute exacerbation of interstitial lung disease at postoperative day (POD) 5, which progressed progressively, and the patient died ultimately at POD 55. In group B, one (1%) mortality occurred within 30 days and two (2%) within 90 days. One patient died of respiratory failure at POD 7 and the other due to lung cancer at POD 65. There was no significant difference between the two groups in terms of death within 30 and 90 days. Figure 4 shows the 5‐year survival rates for groups A and B. The 1‐, 3‐, and 5‐year survival rates for group A were 53.4%, 24.3%, and 24.3%, respectively. The 1‐, 3‐, and 5‐ year survival rates for group B were 89.2%, 69.8%, and 60.0%, respectively.

**TABLE 3:** Comparison of perioperative features between groups A and B.

Variables	Group A	Group B	*p*‐value
	(*n* = 23)	(*n* = 103)	
Operative			
Surgery larger than lobectomy	49 (15%)	4 (5%)	
Lobectomy	176 (55%)	50 (68%)	
Surgery smaller than lobectomy	48 (15%)	10 (13.5%)	0.24^a^
Duration of surgery, min [IQR]	221 [197–341]	146 [125–199]	<0.05
Blood loss, mL [IQR]	180 [30–800]	25 [10–100]	<0.05
Hospital stay, days [IQR]	15 [11–30]	10 [7–14]	<0.05
Postoperative complications	14 (61%)	41 (40%)	0.10
Pulmonary fistula	4 (17%)	6 (6%)	0.08
Arrhythmia	3 (13%)	10 (10%)	0.70
Recurrent nerve palsy	3 (13%)	3 (3%)	<0.05
Chylothorax	2 (9%)	2 (2%)	0.15
30‐day mortality	0 (0%)	1 (1%)	0.52
90‐day mortality	1 (4%)	2 (2%)	0.47

Abbreviation: IQR, interquartile range.

^a^ Surgery larger than lobectomy versus lobectomy and surgery smaller than lobectomy.

## DISCUSSION

We investigated factors predicting aortic invasion in patients with lung cancer. We found that aortic depression and loss of fatty tissue between the tumor and mediastinum were predictive of aortic invasion (odds ratio: 23.86, 16.66). In addition, we found that aortic invasion did not occur in patients with tumor size ≤ 3 cm (0/20). Furthermore, we also did not find aortic invasion in patients in whom the tumor was primarily localized in segment 10 (0/23). Studies have reported that cases of aortic invasion due to lung cancer demonstrate a 10%–50% complication rate after surgery, an approximately 2% death rate within 30 days, and approximately 4% death rate within 90 days, thereby making these surgeries more risky and expensive than regular surgery.^2^, ^3^, ^4^, ^9^, ^10^, ^11^, ^12^, ^13^, ^14^, ^15^, ^16^ Until now, studies have reported methods for determining the presence or absence of mediastinal tumor invasion into the blood vessels, such as CT combined with artificial pneumothorax, dynamic four‐dimensional CT, cine MRI, and respiratory dynamic MRI.^5^, ^6^, ^7^ However, the number of cases in which all of these have been performed is comparatively less and aortic invasion is often overestimated. Glazer et al. reported that tumors appearing to touch the aorta by >90° on chest CT invaded the aorta in four of eight cases, whereas tumors touching the aorta by <90° demonstrated no aortic invasion in any of the 11 cases.^5^ In addition, different specialists, such as surgeons, radiation oncologists, and medical oncologists, have different ways of analyzing it, as they tend to emphasize on radiological findings related to their own specialty. None of the studies till date have discussed tumor localization. However, if the tumor is located specifically in segment 10, it may be less likely to invade the aorta because it is situated farther away from the hilar center in comparison to a tumor located in the upper lobe or segment 6. Moreover, the lower lobe has a wider range of movement due to breathing. Additionally, the lung is a dynamic organ that changes size and shape during the respiratory cycle, which has been shown to reduce the diagnostic accuracy of proximal aortic (aortic arch or ascending aorta) invasion. This is probably a result of limited mobility due to the proximity of the upper lobe to the hilus. Therefore, improving the diagnostic accuracy of proximal aortic invasion will be a challenge. Several studies have reported that chest MRI is better at predicting aortic invasion than chest CT.^6^, ^7^ However, chest MRI has two main drawbacks: (1) longer image acquisition time than chest CT, and (2) the requirement of sufficient supervision of a physician to direct image processing and maintain the quality of examination.

If lung cancer has invaded the aorta, performing radical resection is extremely important from the perspective of prognosis. Shiraishi et al. reported that the 5‐year survival rate for patients in the complete resection group was 36.5%, with two patients surviving for more than 5 years without recurrence, indicating a significantly better prognosis compared to the incomplete resection group.^16^ In cases where the aorta has been invaded, simply removing the adventitia is not sufficient. Moreover, studies have reported that reconstructive surgery has a better prognosis than subadventitial resection.^3^, ^10^, ^14^ In cases where lung cancer has invaded the aortic media or higher level, cardiopulmonary bypass (CPB), cross‐clamp, and endograft procedures are required. In general, aortic resection is associated with higher operative morbidity and mortality when compared with other mediastinal structures, which may correlate with the use of CPB.^4^, ^10^ When using a vascular graft, the occurrence of bronchial stump fistula or empyema can be fatal; thus, some facilities cover the bronchial stump with intercostal muscles or anterior mediastinal adipose tissue with a pedicle. Additionally, although CPB was originally used in several cases, its use has decreased due to the advent of endografts.^17^, ^18^, ^19^ Data regarding the maximum possible extent of aortic resection are lacking. Several studies have reported that no problems occur as long as the resection range is at least 4 cm from the edge of the stent placement site.^19^ There are also reports of placing a synthetic patch, omental flap, xenopericardial patch at the site of the aortic wall defect, but there is no evidence of its usefulness.^19^, ^20^ Moreover, there have been many reports of endograft placement and surgery for lung cancer aortic invasion being performed on the same day.^17^

Opinions are divided as to whether preoperative or postoperative adjuvant therapy should be performed in cases involving the aorta.^21^, ^22^, ^23^, ^24^ We believe that it would be difficult to conduct a controlled study for such specialized surgery. However, it is important to perform contrast‐enhanced CT or PET‐CT before surgery and evaluate the lymph nodes. Studies have also shown that long‐term survival can be achieved in unexpected single N1 and N2 cases.^14^ In addition, there are many reports that N2 cases are diagnosed as systematic disease and that neoadjuvant chemotherapy is recommended first.^11^, ^13^, ^15^ In other words, we believe that tumors with localized lymph nodes are likely to have a different biological behavior than tumors with widespread lymph node metastasis because they have less tendency to metastasize. Therefore, cases in which lymph node enlargement reduces after preoperative chemotherapy may have an improved prognosis. If patients are re‐evaluated using images and N2 positive cases are excluded, we believe that radical surgical resection of the lung tumor with local aortic invasion can be considered.

This study had a few limitations that need consideration. First, this was a retrospective, single‐center study. Moreover, there was no specific protocol and several types of lung cancer cases were included; therefore, the results may not be generalizable. Furthermore, sexuality of the participants was restricted. Second, the selection of cases may have been limited to cases that were originally suitable for surgery. Larger retrospective studies based on protocols from other institutions are needed to validate our results.

In conclusion, we found that no aortic invasion occurred in cases where the tumor was localized to segment 10 or when the tumor was ≤30 mm. In addition, if aortic depression or loss of fatty tissue between the tumor and mediastinum is observed on preoperative chest CT, aortic invasion should be suspected and the surgery should be planned accordingly. However, even if our predictors indicate aortic invasion, patients should still be given the chance to undergo surgery.

## AUTHOR CONTRIBUTIONS

Hideomi Ichinokawa, Kazuya Takamochi, Mariko Fukui, Aritoshi Hattori, Takeshi Matsunaga and Kenji Suzuki conceived and planned the experiments. Hideomi Ichinokawa, Kazuya Takamochi and Aritoshi Hattori planned and performed simulations. Hideomi Ichinokawa, Takeshi Matsunaga and Kenji Suzuki contributed to the sample preparation. Hideomi Ichinokawa, Kazuya Takamochi and Kenji Suzuki interpreted the results. Hideomi Ichinokawa took the lead in writing this manuscript. All authors provided critical feedback and helped shape the research, analysis, and manuscript.

## CONFLICT OF INTEREST STATEMENT

The authors declare no conflict of interest.
